# Trainability of novel person recognition based on brief exposure to form and motion cues

**DOI:** 10.3389/fpsyg.2022.933723

**Published:** 2022-09-28

**Authors:** Kylie Ann Steel, Rachel A. Robbins, Patti Nijhuis

**Affiliations:** ^1^School of Health Science, Western Sydney University, Penrith, NSW, Australia; ^2^The MARCS Institute for Brain, Behaviour and Development, Western Sydney University, Penrith, NSW, Australia; ^3^Research School of Psychology, College of Health and Medicine, Australian National University, Canberra, ACT, Australia

**Keywords:** accuracy, identification, recognition, teammate, video based

## Abstract

Fast and accurate recognition of teammates is crucial in contexts as varied as fast-moving sports, the military, and law enforcement engagements; misrecognition can result in lost scoring opportunities in sport or friendly fire in combat contexts. Initial studies on teammate recognition in sport suggests that athletes are adept at this perceptual ability but still susceptible to errors. The purpose of the current proof-of-concept study was to explore the trainability of teammate recognition from very brief exposure to vision of the whole-body form and motion of a previously unknown individual. Participants were divided into three groups: a 4-week training group who were also the actors for the test and training footage, a 2-week training group, and a no-training group. Findings revealed significant differences between the training groups and their improvement from the pre-to post-test on Response Accuracy and Movement Time. The current study found the best performance in the 4-week Training group. The biggest improvement was found in the 2-week training group, whilst no significant improvement was made in the Control group. These results suggest that training was effective, but also indicate that having initially performed the movements as actors may have led to improvements in baseline testing and ultimately the best results, thus physical performance of skills combined with video-based training may reduce the amount of time needed to improve teammate identification.

## Introduction

When observing human form and motion from bodies and faces, three pieces of information are available to the observer; a person’s identity, their intention, and a subjective emotional response (Dittrich, 1999; Robbins and Coltheart, 2012). Several decades and hundreds of research papers worth of research have investigated face recognition, but more recently body recognition has also started to receive considerable interest. Body form and movement have the advantage of providing information at a distance or when other identifying characteristics, such as the face, are obscured (Yovel and O’Toole, 2016). For example, in crowded high intensity moments in a sports game, players’ faces may be obscured, too far away, or moving too fast to easily identify the person making the body motion and form important cues to identity. In this study we investigate two training paradigms to improve teammate identification from full body form and motion cues.

The human visual system can recognize different forms of motion, and is especially sensitive to the motion created by living organisms (Blake and Shiffrar, 2007). Research suggests this ability is an evolutionary survival trait that developed in early humans allowing them to detect threatening behavior from animals or other humans (Thornton et al., 1998). Early researchers in the human motion field developed point light displays (PLDs), which depict recorded movement from reflective markers. These markers are attached to specific joints on the body and employed to differentiate the relative temporal and spatial information exhibited by movement (Marey, 1895/1972; Johansson, 1973).

The use of PLDs and its variants to study human motion has continued to aid the identification of the specific information that can be attained from seeing motion only including action category, age, deception, emotion, gender, identity, and intention (Johnson and Shiffrar, 2013). PLDs have also allowed the isolation of the relative temporal and spatial cues that allow the identification of specific features. For example, the relationship between the markers on the hips and shoulders for PLD walkers can convey gender with females often showing greater sway between these two body regions (Loula et al., 2005).

These methods and techniques are highly effective in many settings, but others such as sport, benefit from more realistic visual stimuli, especially given the dynamic nature of many sporting activities. In sporting contexts, identification ability is imperative, as it allows teammates to recognize each other and misidentification or a delay in recognition may result in lost scoring or passing opportunities. Passing affordances, for example, stem from classifying players as teammates or opponents (North et al., 2017), and identifying the potential recipient to determine if they are the best tactical option to receive a pass. This is done by differentiating between relevant and irrelevant cues that can be extracted from form, uniform, and biological motion (e.g., players, spectators, and match officials). In a competitive setting with high ball speeds the ball carrier may only have milliseconds to scan the playing field for teammates able to receive a pass (Steel et al., 2010; Hope, 2016). Any inhibition by players in any of these phases of recognition could prove detrimental and cost a team possession of the ball (Steel et al., 2007; Raab et al., 2019; Raab and MacMahon, 2020). Hence developing the ability to quickly identify teammates under stressful match conditions is important for successful performance.

Over the past decade a small number of researchers have applied concepts from the biological motion research field to the examination of teammate identification. This research examines the familiarity of teammates based on human motion and form, and in time constrained environments, in addition to observational learning contexts, for the purpose of improved fundamental and sports skills (Steel et al., 2006, 2008, 2010, 2015; Romeas and Faubert, 2015). This research has shown that members from established sports teams are able to recognize their teammates in short timeframes, including junior and senior invasion sport players who can differentiate between teammates, other players, and non-players from 400 ms video clips of running gait (Steel et al., 2007, 2008) or clips of less than 500 ms of above water swimming gait stimuli (Steel et al., 2006, 2010) and clips of less than 400 ms of ice-skating gait for hockey players (Steel and Dogramaci, 2015). Although scores were significantly above chance, none of the participants in the above research were able to achieve very high levels of recognition performance, despite being members of established teams. The importance of training perceptual-cognitive skills in many sports has been recognized (Hepler and Chase, 2008; Hadlow et al., 2018), especially under time and fatigue constraints (Gabbett et al., 2008), however previous research has not explored the trainability of teammate recognition based on form and motion cues.

Teammate identification is a particularly relevant perceptual-cognitive skill for performance in teams (Hohmann et al., 2011; Romeas and Faubert, 2015), as successful interaction between players may be proportional to their level of familiarity with their teammate’s physical characteristics, movement kinematics, or some combination of both. Familiarity between teammates may particularly affect performance in newly formed representative teams (national or international), where players come together from clubs or squads where they were previously opponents or played in different leagues. Thus, the exploration of the teammate trainability may prove beneficial, particularly between current and recently acquired teammates, to reduce inhibition during the teammate identification process like other perceptual cognitive skills (Hadlow et al., 2018). This may also extend to other team contexts such as the military, law enforcement, or contexts such as crowds where lifeguards and prison guards may need to discriminate their teammates (colleagues) from other populations. Moreover, exploring teammate recognition training may also enhance effective team decisions and actions by increasing the efficacy of shared mental models (SMM). This is where team members’ knowledge, and perception of the factors relative to the context, overlap to enable effective work toward a common goal (Lines et al., 2022).

While the trainability of recognition based on motion and form of whole bodies remains under-researched, some studies have demonstrated promising results when using video or film-based methods. Roark et al. (2006) showed that after watching video of whole-body walking stimuli, participants were able to discriminate familiar actors from non-familiar actors from test footage of moving stimuli of faces when compared to static images of faces, with recognition increasing with repeated views. Further, Robbins and Coltheart (2015), demonstrated that video itself is a viable technique to learn to recognize people from motion. Despite this, neither study examined learning related to sports teams, thus, the potential benefits of increasing this ability in selected contexts alone warrants exploration. Video or film-based training methods may provide an effective learning technique for this purpose, based on their accessibility with current technology. Moreover, video-based methods have been used extensively in perceptual training models within many domains, including sport, with substantial efficacy (Hadlow et al., 2018; Panchuk et al., 2018). For example, Larkin et al. (2018) implemented a video-based decision-making task for Australian Rules football umpires, resulting in improved skills for the intervention group compared to the non-intervention group, with only 20 min training per week. The authors even suggested that the intervention group may have attained reasonably high levels of ability early in training. It is feasible then that firstly, a video-based method of training can improve the perceptual ability associated with teammate identification. Secondly, perceptual improvements can take place with relatively brief periods of training on a limited number of occasions, which is particularly relevant for newly formed representative teams where opponents become teammates, and vice versa for only brief periods of time. Further, designing effective and efficient perceptual training methods that do not increase physical training loads or susceptibility to injury, but consider the demanding schedules of athletes, requires further exploration.

Hence the purpose of this study was to use a video-based method to determine whether teammate identification could be improved, and we hypothesized that (1) a video-based perceptual-cognitive training intervention would improve the accuracy and latency of teammate identification, and (2) a 4-week training program where one group receives weekly training sessions would be better than a 2-week training program where a group only receives two training sessions 3 weeks apart.

## Methodology

### Participants

A total of 38 participants were recruited from the physical education student population at a metropolitan university. Of these, *n* = 12 completed a 4-week video-training intervention (*F* = 4, *M* = 8, 23.9 ± 4.5 years old), *n* = 15 (*F* = 7, *M* = 8, 22.7 ± 3.8 years old) completed a 2-week training intervention, and *n* = 13 (*F* = 5, *M* = 8, 21.7 ± 4.2 years old) were in the control group who received no training. All participants were recruited from the undergraduate human movement programs where the researchers worked, with volunteers self-reporting prior sporting experience that ranged from recreational to club level in both individual and team sports. Participants also reported normal or corrected-to-normal vision, with those who required spectacles or contact lenses asked to wear them during video training sessions. Participants provided written consent prior to taking part in filming and/or training sessions, and ethical approval was granted by the University Human Ethics Committee (Approval Number: H10091). Research was conducted in accordance with the Declaration of Helsinki.

### Design and stimuli

The 4-week training group initially participated as actors to create stimuli, then completed four training/testing sessions across 4 weeks. Each participant saw five people during training. The 2-week training group did not participate as stimulus actors and completed only the first and last of the four training sessions which were the same first and last sessions completed by the 4-week training group. For 4-week and 2-week training groups, each session included a pre-test block, and a training block followed by another test block for each of three different fundamental movement skills—kicking, throwing, and catching. These skills were chosen as they form the basis of most games and sports that individuals learn from an early age and are therefore familiar (Rudd et al., 2015). Further, competent understanding and assessment of these skills occurred during the participants’ studies in human movement, which ensured a suitable level of competency for the actors, and sufficient understanding of the skilled performance by the observers. The control group completed the same testing sessions as the 2-week training group. None of the participants were familiar with the people shown in the stimuli before training. This was checked by presenting examples of the stimulus footage actors to participants at the start of session 1 and asking if they were familiar (yes/no).

**Figure A1:**
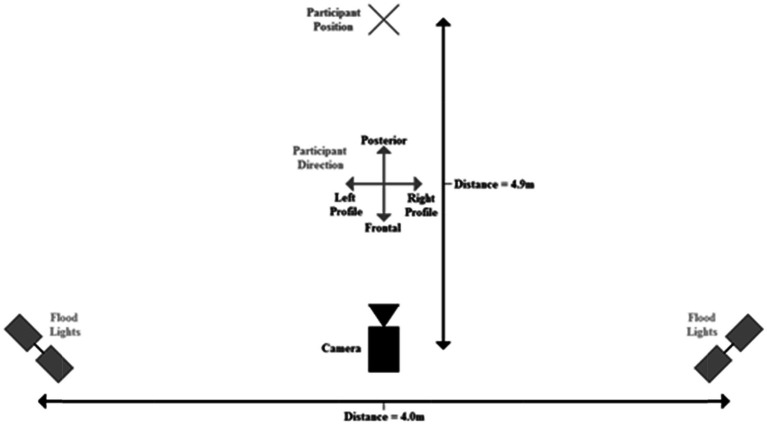
Equipment layout during stimulus collection.

The stimulus actors were filmed individually performing within an indoor sports court at the university of the researchers. The location was kept constant, so each actor performed on the same surface with an identical backdrop. A fixed, tripod mounted, video camera (Sony HDR-FX1000E) was used to capture footage of actors, thus reflecting a first-person viewpoint. To maintain a constant level of lighting, two sets of 2 × 500 watt floodlights, mounted on stands at camera height, were placed adjacent to the camera on either side and aimed at the position where the actors were performing (Figure A1). All actors were young white/white passing adults from a range of gender appearances. These participants were selected to be the actors used in the test footage as they indicated their willingness to complete this task during the recruitment phase of this study. Each actor wore a short sleeve black shirt, black shorts, and sports shoes, with all the additional items such as watches, and hats removed.

Actors were filmed performing three fundamental movement skills from a stationary position: kicking, throwing (two handed chest pass) and catching (two handed) which provided exposure to skills commonly found in most team sports globally. The three skills were performed in four directions with respect to camera position: frontal, posterior, as well as left and right profile (Figure A1). Actors were recorded facing different directions to emulate a sporting context, where interaction among players occurs across a range of viewpoints. Each actor was filmed performing the three skills while facing each direction a total of four times, giving 48 videos of each actor (3 actions × 4 views × 4 repeats). These measures ensured the cues for identification could only be found in the actor’s physical characteristics, movement kinematics, or some combination of both, not in something idiosyncratic about a particular video. That is, we attempted to ensure person identification.

Once filming was complete, the video-based sessions were created using Adobe Premiere Pro CS5 software (Adobe Systems Software, Ltd). Enough stimuli were captured so participants completing video-based training never saw the same trial more than once. Every trial was assigned a number, which was entered into a random sequence generator to determine trial order. All stimuli were 200 ms long, starting at foot/ball and hand/ball contact for kicking and throwing or ending at hand/ball contact for catching. The 2-week training group completed the action blocks in the same order for both sessions.

### Procedure and apparatus

Participants were briefed at the beginning of each training session with an outline of the task, the components involved, and when and how to respond. In addition to session briefings prior to training, on-screen instructions were displayed at the beginning of each new block of testing and training. Further, participants viewed a familiarization clip prior to the first testing and training session to ensure they were aware of the brevity of each clip.

Each session began with a test block; in the first session this was to confirm that participants did not have prior knowledge of the actors, and always included previously unseen stimuli of all three skills. Each training (or familiarization) session included three training blocks—one for each skill—each of which was immediately followed by a testing block. Each session was 12 min in duration which is reflective of the short time availability of many athletes due to their numerous training and daily commitments. No feedback was provided to participants in any of the three groups in this study.

**Figure 1:**
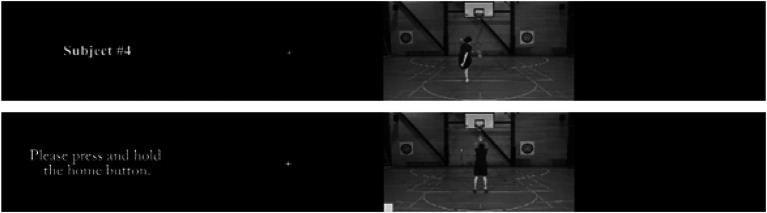
Examples of training (top) and testing (bottom) trial sequences.

On each trial of the training blocks, participants were presented first with the actor number (e.g., #1) for 2 s, then a fixation cross for 2 s, followed by the video of the actor performing a skill for 200 ms, then a black screen for 2 s (Figure 1). The training block for each skill was immediately followed by the testing block for that skill. On each trial of the testing block the instruction “please press and hold the home button” slide was presented for 2 s, followed by a central fixation cross for 2 s. Both slides served to prepare the participant for the subsequent video of a subject performing a skill for 200 ms, and then a black response screen for 5 s during which the response could take place (Figure 1). After the initial test block to determine baseline ability and ensure the actors were not known to the observers, participants were instructed to consider each actor in a training block as a teammate. Specifically, they had to indicate whether the actors in the subsequent test block were a teammate (familiar), or non-teammate (unfamiliar: not present in the preceding training block). Participants could respond sooner if they chose to though the clips were so brief that no participants processed this information quickly enough to respond before the black screen.

Each block (training/testing) contained 10 trials, which showed 0–4 examples of each of the actors performing one skill (e.g., kicking), facing any of the four directions (frontal, posterior, as well as left and right profile). The number of trials per actor was not equal so that participants could not guess the actor in the later trials based on who they had already seen in earlier trials. Training and test blocks could contain a mix of movement skills.

Training sessions were conducted on either a desktop computer or laptop with similar screen sizes. Viewing height was at eye level and viewing distance was 1 m from participants. Data in testing blocks was collected using a purpose-built latency apparatus capable of millisecond accuracy, and custom designed software permitted connectivity between the latency apparatus and computer (Steel and Eisenhuth, 2012). The latency apparatus consisted of a home button toward the bottom center of the device, and six response keys arranged in a semi-circular pattern toward the top. The response keys were arranged in this fashion, so they were at an equal distance from the home button. Buttons were labeled 1–5 from left to right respectively, with the right most button not being used in this experiment.

### Dependent variables and statistical analysis

Performance in this study was measured as the percentage of correct identification decisions where response accuracy (RA) indicated the participant’s ability to correctly identify actors present in the associated training block. In addition, we measured the average time taken to respond in testing blocks, response time, separated in reaction time (RT), and movement time (MT). RT was measured as the time between a stimulus clip starting and the participant physically initiating a response, whereas MT was the time between physically initiating a response and selecting a response. Even though the 4-week training group had a total of four sessions, comparisons between groups were made based on the first and last session, since these were performed by all groups. Each dependent variable was analyzed individually using 3 (Training Group) x 2 (Session) x 3 (Block) mixed model ANOVAs, with Training Group as a between-subjects factor and Session and Block as within-subjects factors. The 4-week training group was additionally analyzed with a 4 (Session) x 3 (Block) repeated measures ANOVA and a 4 (Session) x 3 (Skill) repeated measures ANOVA. Statistical analyses were performed using JASP 0.12.2.0 (open source https://jasp-stats.org/).

## Results

### Response accuracy

**Figure 2:**
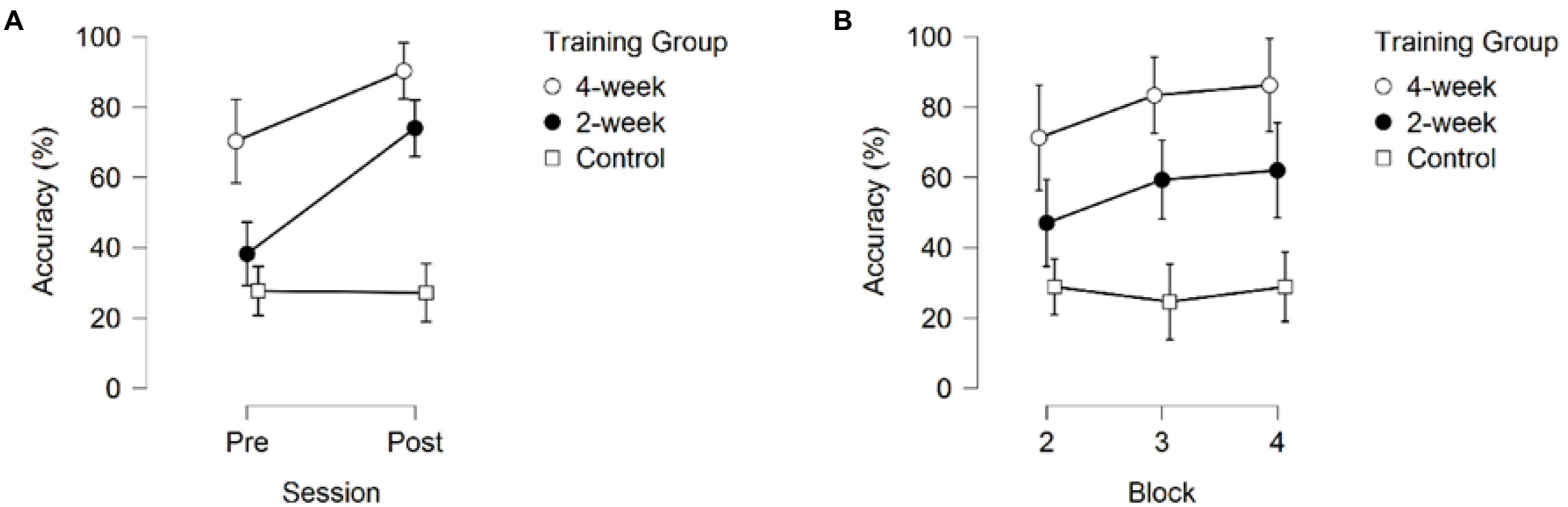
The Response Accuracy **(A)** across sessions, averaged over the three blocks **(B)** across blocks, averaged over the pre- and post-session. Error bars represent 95% CIs between groups.

A 3 (Training Group) × 2 (Session) × 3 (Block) mixed model ANOVA returned significant main effects for Training Group *F*(2,37) = 21.607, *p* < 0.001, partial *η*^2^ = 0.539, Session *F*(1, 37) = 22.883, *p* < 0.001, partial *η*^2^ = 0.38 and Block, *F*(2, 74) = 7.970, *p* < 0.001, partial *η*^2^ = 0.177. Additionally, a significant interaction between Session and Training Group, *F*(2, 37) = 7.805, *p* = 0.0012, partial *η*^2^ = 0.297, and an interaction between Block and Training group, *F*(2, 37) = 2.815, *p* = 0.031, partial *η*^2^ = 0.132, were found (Figure 2).

**Figure 3:**
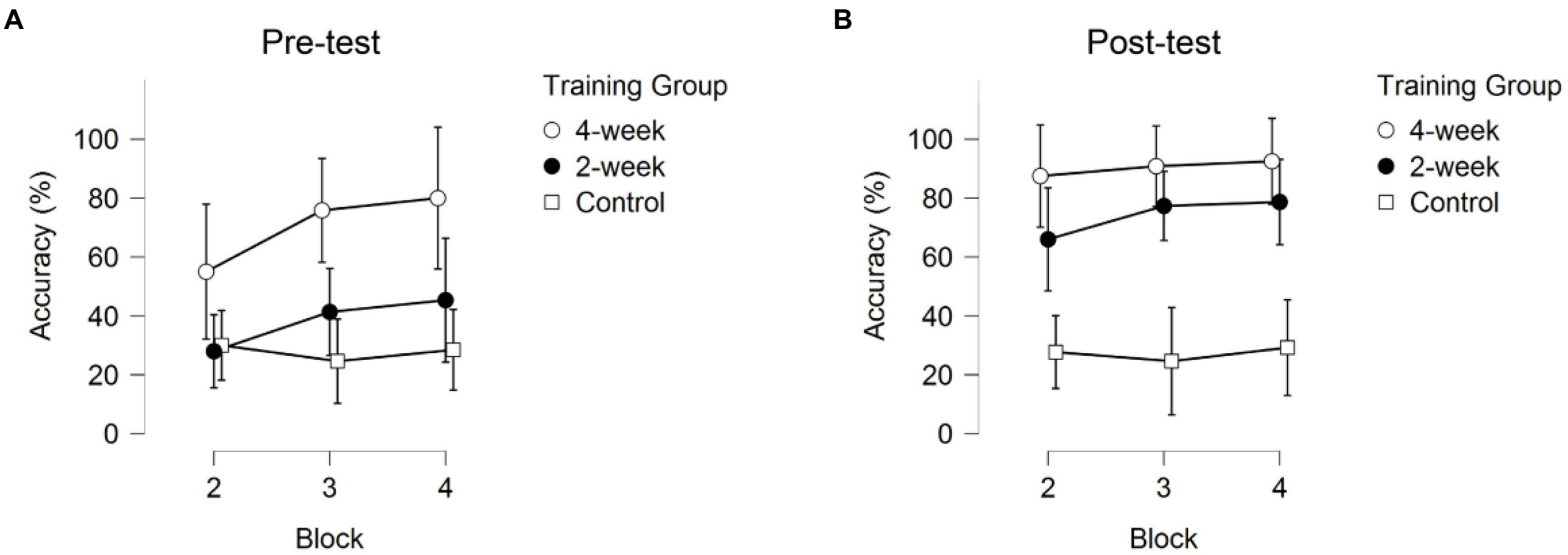
The Response Accuracy across the three blocks for the pre-test and post-test sessions. Error bars represent 95% CIs between groups. **(A)** after the term “pre-test” and **(B)** after “post-test”.

*Post-hoc* pairwise comparisons for Response Accuracy averaged across sessions and blocks revealed significant differences between the 4-week training group, the 2-week training group, and the no-training group. As depicted in Figure 2 the 4-week training group correctly identified teammates significantly more often than the 2-week training group (*p*_bonf_ = 0.011) and the no-training group (*p*_bonf_ < 0.001). Additionally, the 2-week training group correctly identified teammates significantly more often than the no-training group (*p*_bonf_ = 0.002). Post-hoc comparison for Session (averaged across training groups and blocks) showed higher Response Accuracy during the post-test than during the pre-test (*p* < 0.001), see Figure 3A.

Post-hoc comparisons of the significant interaction between Session and Training group (*p* = 0.001) showed that the 2-week training group was the only group showing robust significant improvement in Response Accuracy between the pre-and post-sessions (*p*_bonf_ < 0.001). The 4-week training and no-training group did not show significant improvement in Response Accuracy between the pre-and post-sessions (*p*_bonf_ = 0.105, *p* = 0.007, *p*_bonf_ = 1.000, *p* = 0.940 respectively). It is important to note that the 4-week training group already scored significantly higher than the 2-week training group (*p*_bonf_ = 0.012, *p* < 0.001) and the no-training group (*p*_bonf_ < 0.001, *p* < 0.001) during the first session (pre-test) without receiving any training, whereas the 2-week training and no-training group did not significantly differ in their initial RA performance (*p*_bonf_ = 1.000, *p* = 0.242). The 4-week training group also did not significantly differ from the 2-week training group on the post-test (*p*_bonf_ = 1.000, *p* = 0.079), but both the 4-week training and 2-week training group correctly identified teammates significantly more often than the no-training group on the post-test (*p_bonf_* < 0.001, *p* < 0.001; see Figure 2A).

Pairwise comparisons (averaged across training groups and sessions) revealed significant differences between block 2 and 3 (*p*_bonf_ = 0.031, *p* = 0.010), block 2 and 4 (*p*_bonf_ < 0.001, *p* < 0.001), but not between block 2 and 3 (*p*_bonf_ = 0.613; see Figure 2B). This indicates a ceiling effect where participants’ familiarity with actors reached a point where no further improvement was possible under the current design.

The significant interaction between Training Group and Block indicated that the three groups showed different progressions during a training session. Only the 2-week training group showed significant improvement, and only between block 2 (kicking) and block 4 (throwing; *p*_bonf_ = 0.020). The 4-week training and no-training group did not show significant improvement (*p*_bonf_ = 1.000). As can be seen in Figure 3, the 4-week training group did improve, although not significantly, between blocks in the first session (pre-test). No further improvement was seen for the 4-week training group in the post-test, indicating performance had already stabilized in the previous three sessions.

### Reaction time

**Figure 4:**
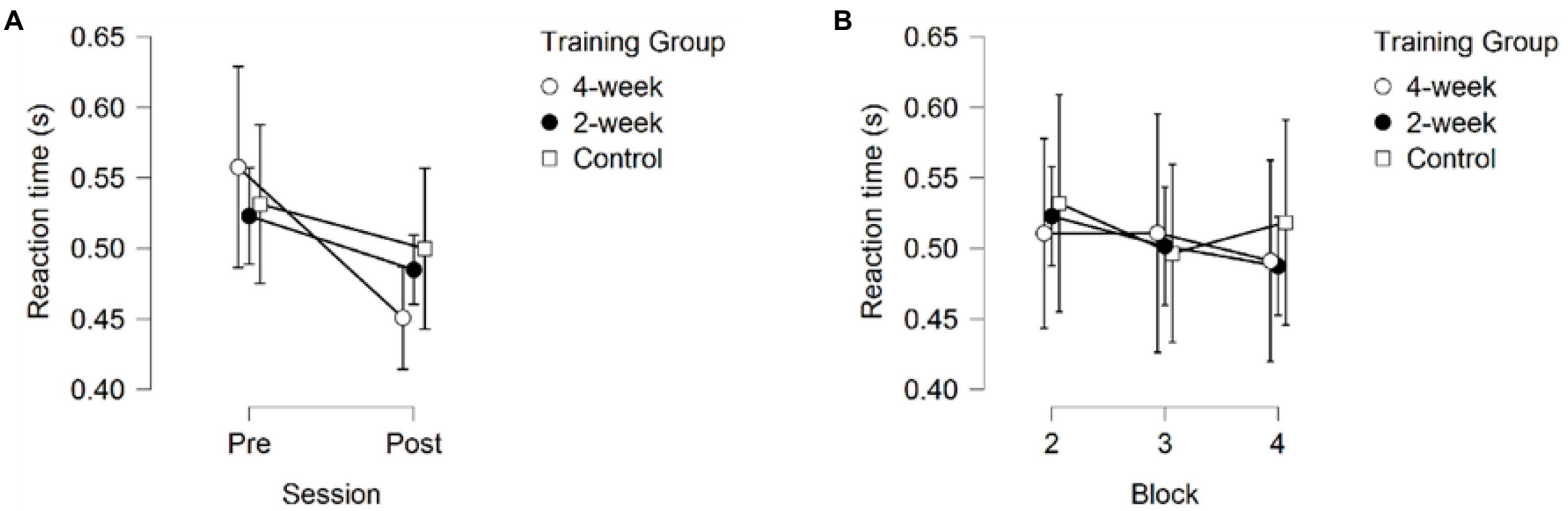
Reaction Time **(A)** across sessions, averaged over the three blocks **(B)** across blocks, averaged over the pre- and post-session. Error bars represent 95% CIs between groups.

A 3 (Training Group) × 2 (Session) × 3 (Block) mixed model ANOVA on Reaction Time (RT) only returned a significant main effect of Session *F*(1, 37) = 5.355, *p* = 0.026, partial *η*^2^ = 0.126, showing that all participants got faster at initiating responses, see Figure 4. No significant main effects of Training Group *F*(2,37) = 0.043, *p* = 0.958, partial *η*^2^ = 0.002, or Block *F*(1.478, 54.689) = 2.798, *p* = 0.085, partial *η*^2^ = 0.070. No significant interactions were observed, *p* > 0.407.

### Movement time

A 3 (Training Group) × 2 (Session) × 3 (Block) mixed model ANOVA on Movement Time (MT) returned significant main effects for Training Group *F*(2,37) = 8.859, *p* < 0.001, partial *η*^2^ = 0.324, Session *F*(1, 37) = 49.419, *p* < 0.001, partial *η*^2^ = 0.572, and Block *F*(1.657, 61.322) = 13.291, *p* < 0.001, partial *η*^2^ = 0.264. Additionally, a significant interaction between Session and Training Group was found, *F*(2, 37) = 3.787, *p* = 0.032, partial *η*^2^ = 0.170.

**Figure 5:**
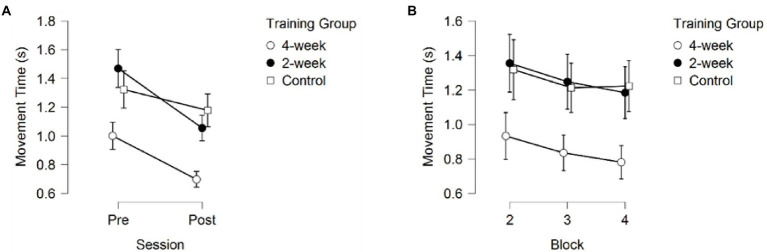
Movement time **(A)** across sessions, averaged over the three blocks **(B)** across blocks, averaged over the pre- and post-session. Error bars represent 95% CIs between groups.

*Post-hoc* pairwise comparisons for Movement Time collapsed across blocks and sessions revealed significantly faster movements for the 4-week training group compared to the 2-week training group (*p*_bonf_ = 0.002), and the no-training group (*p*_bonf_ = 0.003), suggesting faster decision making, but no differences in MT between the 2-week training group and the no-training group (*p*_bonf_ = 1.000), as depicted in Figure 5.

**Figure 6:**
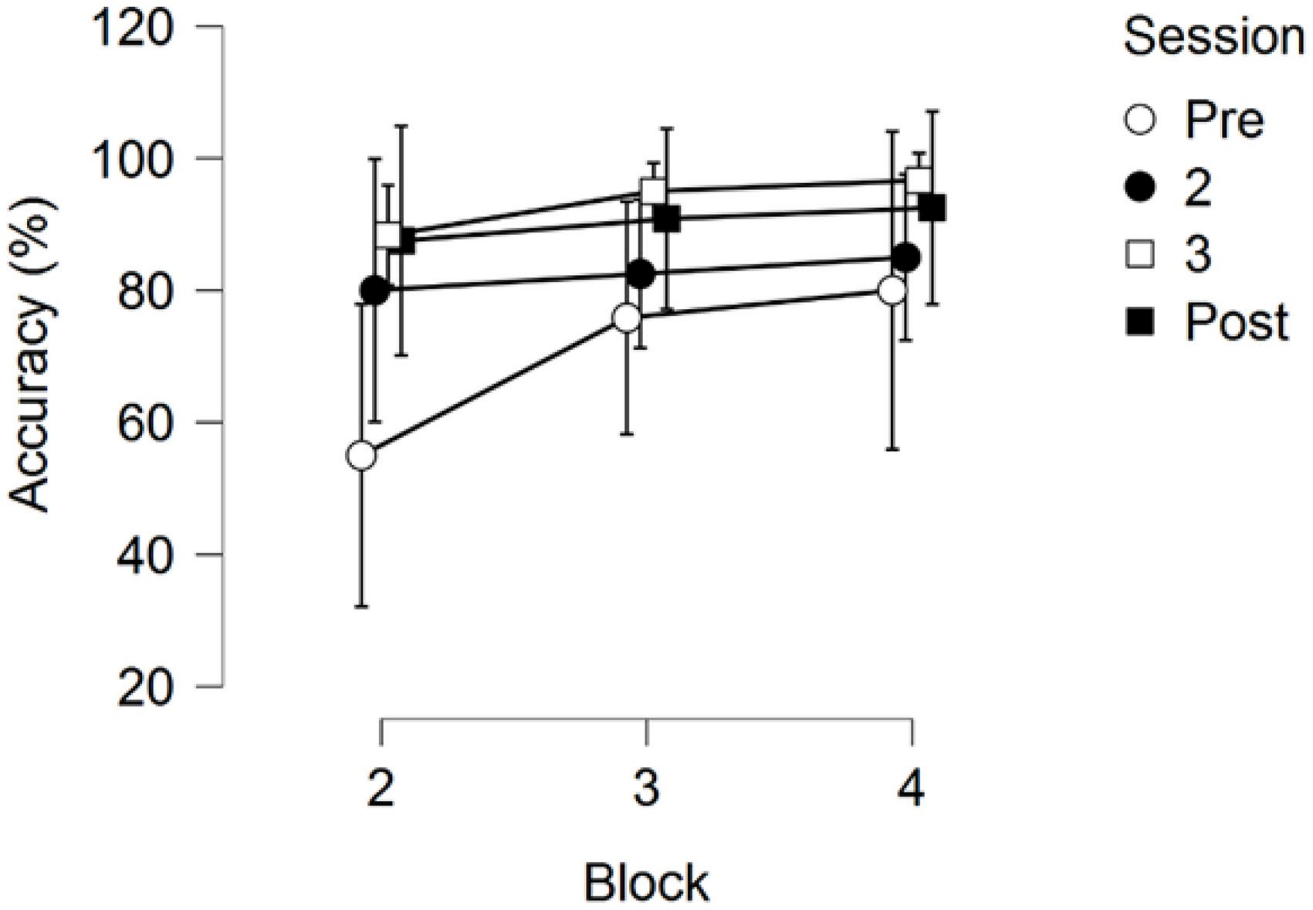
Response Accuracy across the three testing blocks for the four training sessions. Error bars represent 95% CIs.

The main effect for Session showed faster MT during the post-test than during the pre-test (*p* < 0.001), see Figure 5A. Additionally, post-hoc comparisons of the significant interaction between Session and Training group (*p* = 0.032) showed significant improvement in MT between the pre-test and post-test for the 4-week training group (*p*_bonf_ = 0.004) and the 2-week training group (*p*_bonf_ < 0.001), but not for the no-training group (*p*_bonf_ = 0.727). It is important to note that the 4-week training group already moved significantly faster than the 2-week training group (*p*_bonf_ = 0.004, *p <* 0.001) but did not move significantly faster than the no-training group (*p*_bonf_ = 0.176, *p* = 0.012) during the first session (pre-test) and even the very first training block (*p*_bonf_ = 0.037 and *p*_bonf_ = 0.071, respectively) without receiving any prior training, whereas the 2-week training and no-training groups did not significantly differ in their MT during the first session (pre-test; *p*_bonf_ = 1.000, *p* = 0.219). The 4-week training group also did not significantly differ from the 2-week training group on the post-test (*p*_bonf_ = 0.064, *p* = 0.004), but did move significantly faster than the no-training group (*p*_bonf_ = 0.004, *p* < 0.001) on the post-test, whereas no difference in MT was found between the 2-week training group and the control group (*p*_bonf_ = 1.000, *p* = 0.300; see Figure 6).

Pairwise comparisons on the main effect of Block on MT revealed significant differences between block 2 and 3(*p*_bonf_ = 0.001, *p* < 0.001), block 2 and 4 (*p*_bonf_ < 0.001, *p* < 0.001), but not between block 3 and 4 (*p*_bonf_ = 0.617, *p* = 0.206; see Figure 5B). These results are consistent with the effects observed in Response Accuracy.

### Four-week training group: All training sessions

#### Response accuracy

A 4 (Session) × 3 (Block) repeated measures ANOVA on Response Accuracy (RA) returned significant main effects for Session *F*(3,33) = 4.558, *p* = 0.009, partial *η*^2^ = 0.293, and Block *F*(2,22) = 8.367, *p* = 0.002, partial *η*^2^ = 0.432. No interaction between Session and Block was found, *F*(2.636, 28.995) = 1.340, *p* = 0.280, partial *η*^2^ = 0.109; see Figure 6.

Pairwise comparisons of the main effect of Session showed that the first training session showed significantly lower RA than the third (*p*_bonf_ = 0.011) and the fourth session (*p*_bonf_ = 0.036). No significant differences were found between the third and the fourth session (*p*_bonf_ = 1.000), nor were any significant differences found between the second training session and the other three training sessions (all *p*-values > 0.05).

Pairwise comparisons of the factor block found that the second block (first skill) had significantly lower RA than the following third (*p*_bonf_ = 0.020) and fourth block (*p*_bonf_ = 0.002), but no difference in RA between the third and fourth block was observed (*p*_bonf_ = 1.000).

#### Reaction time

**Figure 7:**
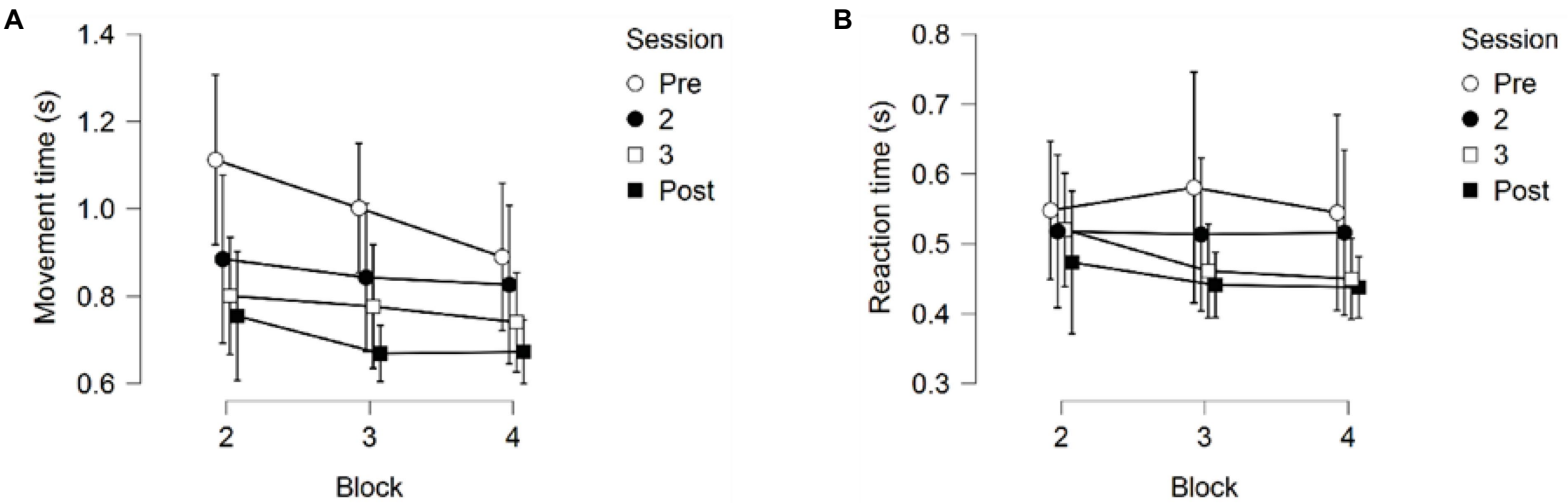
**(A)** Reaction time for the four training sessions across the three testing blocks. **(B)** Movement time for the four training sessions across the three testing blocks. Error bars represent 95% CIs between groups.

A 4 (Session) × 3 (Block) repeated measures ANOVA on Reaction Time (RT) returned no significant main effects for Session, *F*(1.852,20.376) = 1.506, *p* = 0.245, partial *η*^2^ = 0.120, or Block, *F*(2,22) = 3.032, *p* = 0.069, partial *η*^2^ = 0.216. No interaction between Session and Block was found either, *F*(2.977, 32.748) = 2.101, *p* = 0.120, partial *η*^2^ = 0.160, see Figure 7B.

#### Movement time

A 4 (Session) × 3 (Block) repeated measures ANOVA on Movement time (MT) returned significant main effects for Session *F*(1.860,20.463) = 7.927, *p* = 0.003, partial *η*^2^ = 0.419, and Block *F*(2, 22) = 15.633, *p* < 0.001, partial *η*^2^ = 0.587; see Figure 7A. No significant interaction between Session and Block was found, *F*(2.457, 27.022) = 1.444, *p* = 0.254, partial *η*^2^ = 0.116.

Pairwise comparisons for the main effect of Session showed that the first training session showed significantly slower MT than the third (*p*_bonf_ = 0.008) and the fourth session (*p*_bonf_ < 0.001). No significant differences were found between the third and the fourth session (*p*_bonf_ = 1.000), nor were any significant differences found between the second training session and the other three training sessions (all *p*-values > 0.05).

Pairwise comparisons for the main effect of block found that the second block (first skill) had significantly slower MT than the following third (*p*_bonf_ = 0.007) and fourth block (*p*_bonf_ < 0.001), but no difference in MT between the third and fourth block was observed (*p*_bonf_ = 0.140).

## Discussion

The purpose of this proof-of-concept study was to examine the efficacy of a video-based training method for teammate recognition based on form and motion cues. The results showed that the 4-week training group, who also participated as actors, performed significantly better compared to the 2-week and no-training groups, while the 2-week training group performed significantly better overall than the no-training group. However, the 2-week training group was the only group to significantly improve from pre-test to post-test, despite performing worse than the 4-week training group on the post-test. Further, both training groups were significantly more accurate than the no-training control group at the post-test showing that training did occur. Most improvement occurred between the first two blocks of training (blocks 2 and 3, given that block one was the familiarity test). Thus, these results support the concept of trainability in whole-body video-based teammate identification.

Both training groups showed significant improvement in Movement Time from pre-to post-test, indicating that training enabled participants to act faster. At the post-test, the 4-week training group showed faster movement times than the other two groups, while both training groups had significantly faster movement times than the no-training group, but not each other, suggesting improvement in movement speed was due to training, rather than familiarization with the experimental task.

In contrast, a significant overall improvement from pre-to post-test in Reaction Time was also found, but no differences between the groups were found, suggesting Reaction Time improved with familiarization of the task rather than a training effect.

A difficulty with any training study is to match all experimental groups pre-training so that any differences at post-training can only be attributed to training. Unfortunately, groups were not matched on skill at the commencement of the study leaving open the possibility that the 4-week training group were just better overall at recognizing people. Although we cannot completely rule this out, there was another interesting difference between the two training groups—the 4-week training group had participated in the movements as part of the stimulus creation. Performing and familiarization with the kinematics of the tested movements may explain the baseline benefit of the 4-week training group.

Moreover, the observation of movement is “contagious” in that it activates the motor system in its observer, in particular the Action Observation Network (AON); a neural network that has been shown active in response to other agents’ actions (Buccino et al., 2004). These motor simulations in response to others’ actions help understand and predict others’ behavior and their outcomes. Previous research demonstrates greater AON activity when watching visually or physically familiar actions (Buccino et al., 2004; Calvo-Merino et al., 2005; Cross et al., 2006; Press, 2011). Furthermore, Kirsch and Cross (2015) showed that training multimodal aspects, including the physical aspects of dance movements resulted in stronger neural responses in the AON for participants who scored better while performing the observed action (Kirsch and Cross, 2015). These results suggest that familiarity with and skill in the execution of movements strengthens the neural response to, and understanding of, such movements (Gardner et al., 2015). In relation to the current study, the majority of the 4-week training group (10/12) performed the skills for the recording of stimuli, hence they were more familiar with the movements as they had physically performed the movements. For these participants, increased engagement of the AON and their ability to simulate the movements might explain their initial advantage in the pre-test and underlines the role of performing the movements (kinematics) one is tasked to recognize.

Another possible concern is the small number of stimuli (i.e., players) each participant was exposed to during training. We deliberately chose five as a number similar to the approximate number of players for some team sports (e.g., 4–6 players for basketball, futsal, indoor hockey), but which would be unlikely to result in chance performance, as might be found for larger numbers of players. Indeed, the control group performance was at chance, but this is to be expected given that they received no feedback as to the identifiers of the stimuli. Training groups outperformed chance levels easily. In the future, additional confidence ratings could be collected following each trial (i.e., was the participant certain or guessing?; *cf*., Steel et al., 2006, 2007, 2008), however, we wanted to keep training sessions brief to emulate the already time poor training schedules of athletes (Larkin et al., 2018). Our current proof-of-concept suggests that these short sessions were useful, though perhaps most useful when the participants had already performed the tested movements. In the future, replication of the current findings with a larger number of stimuli, including considerations of interchange or substitution, should be considered to investigate the impact of memory load on teammate recognition training as well as more systematic testing of the role of familiarity with movement kinematics of the tested skills.

While this study demonstrated improvements in the accuracy and latency of TM-ID, the visual cues used for identification are still unknown. There were no quantitative or qualitative measures employed to discover what cues participants relied upon for identification, whether they were physical characteristics, movement kinematics, or some combination of both (Steel et al., 2010; Robbins and Coltheart, 2012). These cues might be particularly relevant in a more cluttered visual field that is present during game scenarios (i.e., 22 players on a soccer field), which was not tested in this study. Future research should attempt to capture the cues used for identification in sporting contexts by recording the eye movements of participants while observing more ecologically valid stimuli (de'Sperati and Stucchi, 1995). This was beyond the scope of the current proof-of-concept study.

The findings of this research could be applied to newly formed representative sporting teams to determine whether perceptual-cognitive training would benefit on-field performance. Improvements in teammate identification may lead to faster and more accurate decision making, more efficient movement, as well as enhanced performance outcomes such as passing speed, completion rates, and tactical play. Future studies could also expand upon the current proof-of-concept study by incorporating team development interventions (TDI) that consider the impact of shared mental models (Lines et al., 2022). This would be particularly important if variables such as ability, attitude, and beliefs influence perception of new team members.

This study has established that as little as two training sessions over 4-weeks elicits significant improvements in TM-ID with respect to accuracy and latency. Further, the duration of training sessions is short enough so regular match preparation would be unaffected, and it could be integrated into current training schedules.

Given the proliferation of digital and mobile technology, TM-ID training could take place outside of the laboratory, such as in dressing rooms, to be integrated with physical training. Moreover, the current paradigm could be applied to more cluttered visual fields and used to extract visual cues relevant to teammate identification. Further research is needed to answer these questions and determine whether perceptual-cognitive training of this nature translates to in-game performance. Finally, while combining physical execution and observation are effective for movement skill learning (Wulf et al., 2010), its impact on perception of biological motion is unclear. Thus, future research could explore the impact of combined visual and physical learning on perception and could be achieved by comparing novices and experts.

## Conclusion

Video-based training is an appropriate method to improve the accuracy and latency of teammate identification (TM-ID). Results indicate participants were able to significantly improve their performance, demonstrated by increases in response accuracy (RA), as well as decreases in reaction time (RT) and movement time (MT). Further, it was more beneficial to complete the full training program, which included performing the movements. These findings demonstrate the benefit of video-based perceptual-cognitive training on teammate identification in team invasion sports. For a player, being able to quickly and confidently identify a teammate may influence other aspects of the game, even the outcome. Ultimately, this indicates the specialized training experienced by participants here could be employed in newly formed representative teams where players may be unfamiliar with the identity of their new teammates (Steel et al., 2010).

## Data availability statement

The raw data supporting the conclusions of this article will be made available by the authors, without undue reservation.

## Ethics statement

The studies involving human participants were reviewed and approved by Western Sydney University Human Research Ethics Committee. The patients/participants provided their written informed consent to participate in this study.

## Author contributions

KS and RR were involved in all aspects of the project, while PN was actively involved in data analysis and manuscript development. All authors contributed to the article and approved the submitted version.

## Conflict of interest

The authors declare that the research was conducted in the absence of any commercial or financial relationships that could be construed as a potential conflict of interest.

## Publisher’s note

All claims expressed in this article are solely those of the authors and do not necessarily represent those of their affiliated organizations, or those of the publisher, the editors and the reviewers. Any product that may be evaluated in this article, or claim that may be made by its manufacturer, is not guaranteed or endorsed by the publisher.
